# Supplementary material for: Are carbon emissions trading and green financial instruments synergistic? -Comprehensive quantitative research based on content analysis
Source: PLoS One. 2024 Mar 7;19(3):e0298601. doi: 10.1371/journal.pone.0298601 (PMC10919601; doi:10.1371/journal.pone.0298601)
Supplement: S1 Data — (ZIP) [file pone.0298601.s001.zip › Data/地级市Z_F工作报告.zip!/╕ú╜¿/─■╡┬╩╨/└┤╫╘╣1⁄2╓┌║┼í╛┬φ┐╦╩2╛▌═°í┐_2010.docx]

各位代表：

现在，我代表市人民政府向大会报告政府工作，请予审议，并请市政协各位委员和其他列席人员提出意见。

一、2009年的主要工作

过去的一年，在省委、省政府和市委的坚强领导下，全市各级政府全面贯彻党的十七大、十七届三中、四中全会精神，深入学习实践科学发展观，认真落实国务院支持福建省加快建设海峡西岸经济区的《意见》，坚决实施中央和省应对国际金融危机的一揽子计划，全力保增长、保民生、保稳定，加快推进环三都澳区域发展和海西东北翼中心城市建设，经济社会发展取得新的成效。全市生产总值604亿元，增长13.3%；财政总收入45亿元，增长11.7%，其中，地方级财政收入28亿元，增长13.7%；全社会固定资产投资287亿元，增长21%；外贸出口总额下降15.1%；验资口径实际利用外商直接投资5672万美元，增长48.7%；社会消费品零售总额228亿元，增长14%，居民消费价格总水平下降1.1%；城镇居民人均可支配收入15147元，实际增长11.4%；农民人均纯收入5838元，实际增长8.8%；城镇登记失业率4%；人口自然增长率6.3‰。单位生产总值能耗降低0.7%，非电二氧化硫排放量减少2.75%，化学需氧量排放与上年持平。一年来的主要工作和成效是：

（一）把握机遇乘势而上，环三开发扎实推进

研究制定我市贯彻落实国务院《意见》的实施意见，进一步完善和明确环三发展的功能定位、发展布局和目标任务，重点在政策创新、项目布点、交通建设、产业集聚、宁台合作等方面跟进衔接，及时细化分解相关任务，落实责任，推动环三发展更好地融入海西建设大局。着力构建环三发展规划体系，与环三总体规划相配套的专项规划编制或修编基本完成。上级支持环三发展力度加大，已有17个省直部门出台支持措施。周边地区与环三区域的协作更加紧密，江西上饶市与我市签订共同建设出海通道、加强旅游交流合作框架协议，浙江丽水市、衢州市也就合作事宜与我市达成初步协议。启动区溪南半岛开发扎实推进，海西宁德工业区管委会正式挂牌成立，去年12月工业区正式开工建设。大唐国际发电股份有限公司与我市签订《关于在宁德投资建设项目的合作框架协议》，双方就共同推进火电、风电、核电、潮汐能电站、煤炭集散中心、白马港铁路支线等项目达成合作意向。宁德核电一期工程加快建设，累计完成投资130多亿元。大唐火电二期两台机组投产运营，华能核电、聚合物锂离子电池、镍合金、大型钢铁、海上风电、大型造船、豪华游艇、精细化工等一批在建在谈项目加快推进。在宁德投洽会上，环三区域签约项目82个，总投资183亿元，占全市签约项目投资总额的60.2%。

（二）有效应对国际金融危机，经济持续较快增长

农业农村稳步发展。农林牧渔业总产值196亿元，增长6.1%。各项强农惠农政策有效落实，粮播面积和粮食产量保持稳定。茶叶、食用菌、水产、林竹等主导产业不断发展壮大，建成国家级农业生产示范区3个、省级示范区12个，省级以上农业产业化重点龙头企业实现销售收入29亿元，增长18.3%。福安、福鼎入选“全国十大产茶县”，霞浦被评为“中国海带之乡”、“中国紫菜之乡” 。坦洋工夫、福鼎白茶被评为中国驰名商标。福鼎白茶、蕉城天山绿茶、霞浦海带、霞浦紫菜经核准注册为地理标志证明商标，全市地理标志证明商标达18个，居全国各设区市首位。社会主义新农村试点示范村建设扎实推进。农村基础设施逐步改善，实施土地治理4.8万亩，修复病险水库11座，完成海堤除险加固工程25.5公里、农村公路“上衔下延”工程348公里，建成危桥改造项目40个、农村客运站7个、农村候车亭160个，完成17个乡镇429个村垃圾治理任务，新建农村户用沼气池9500口。动植物疫情疫病防控切实加强，防抗“莫拉克”台风等自然灾害扎实有力，因灾倒房户春节前全部搬入新居。

工业经济较快增长。全年工业总产值738亿元，增长20.1%，增加值204亿元，增长16%。其中，规模以上工业总产值617亿元，增长22.7%，增加值164亿元，增长19.8%，两项增幅连续五年居全省设区市首位，工业总量上升到全省设区市第8位。新增规模以上企业108家，实施省工业制造业重点项目22项，完成工业投资150亿元，增长41.3%。国有及国有控股企业实现工业总产值83亿元，增长15.9%，主营业务收入65亿元，增长15.4%，利税总额8.5亿元，增长569.2%，闽东电力利润增长近1倍，创上市以来最高。福安、福鼎分别被授予“中国中小电机之都”、“中国化油器名城”称号。福安亚南电机、福鼎大吉剪刀被评为中国驰名商标，29家企业获得省著名商标，19种产品获得省级名牌产品称号。

投资拉动强劲有力。“项目建设攻坚年”活动扎实推进，100个重点项目完成投资151亿元，增长22%，投资对经济增长的贡献率达73.4%，提高20.8个百分点。市财政投入项目前期工作专项经费1700万元，有力推动了重大项目前期工作的深入开展。温福铁路正式开行动车组，宁德率先进入海西高铁时代。宁武高速公路宁德段全线动工，白马港铁路支线开工建设。104国道改造全面完成，宁古二级公路、霞浦盐田至溪南疏港二级公路以及4个5000吨级码头项目加快推进。积极争取前四批中央新增投资项目802项、总投资38.9亿元，其中列入国家金宏网管理项目220项、总投资20.6亿元，已竣工项目82个。

消费需求持续升温。旅游消费不断增长，全年接待游客突破600万人次，旅游收入46.4亿元，增长10.3%。宁德世界地质公园申报迎检工作有效推进，白云山景区被评为国家级风景名胜区。全市商品房销售面积159万平方米，增长132.8%。汽车成为新的消费热点，全市汽车销售额2.8亿元，增长57.9%。中国海峡大茶都、古田食用菌批发市场项目列入商务部“双百市场工程”，东方伟业沃尔玛购物广场完成主体工程。改造农贸市场17个，建成生猪、蔬菜等副食品基地40个，“万村千乡市场工程”配送中心4个、农家店421个。家电、摩托车下乡和汽车以旧换新扎实推进，全市家电下乡销售额1.8亿元。产品质量和食品药品安全专项整治深入开展，市场物价保持稳定。

城市建设步伐加快。以承办第三届茶博会为契机，实施一批市政基础设施建设，会展中心扩建工程、五星级会展酒店一期工程、宁德火车站站房和站前广场一期工程等城市配套服务设施，东湖北岸景观公园、人民广场等城市景观设施，高速公路宁德南北互通口扩建工程、福宁路一期工程以及万安路、华庭路、尚德路、金马路、塔山路等市政道路相继建成投入使用，东湖清淤工程扎实推进，水、电、广播电视、通讯、互联网等全面覆盖新城区。城市卫生、园林绿化、户外广告、建筑工地、集贸市场等专项整治深入推进，市容市貌明显改善，宁德中心城市形象进一步提升。

改革开放不断深化。农村综合改革扎实推进，集体林权、海域和内陆水域使用权、小型农田水利设施产权等各项改革取得明显成效。国有资产监督管理体系逐步完善，财政、流通、城市管理以及教育、科技、文化等领域的体制改革不断深化。福建海峡银行宁德分行正式开业。三都澳水域开放取得实质性进展。与马来西亚诗巫市缔结友好城市，与美国、英国、德国的4个城市签署友城意向。招商引资成效明显，宁德投洽会共签约合同项目158个、投资总额204亿元，履约率达75.9%。积极落实稳定外贸增长的各项政策措施，船舶、水产等重点行业出口保持增长，新增外贸资格备案企业96家。宁台交流合作更加紧密。第三届海峡两岸茶业博览会成功举办，共有300多名台湾嘉宾到会，73家台湾茶企100多个展位参展，创下台湾本土茶企赴大陆参加茶业展会新纪录。展会共签约投资、购销等合同127个、总金额111亿元，其中台资3685万美元。先后3次组团赴台开展交流及推介宁德活动，陈靖姑金身首次巡游台湾和甘国宝诞辰300周年纪念活动圆满成功。宁德—基隆经济文化对口合作协调处挂牌成立，台湾嘉义县阿里山乡和南投县竹山镇分别与福鼎点头镇、福安社口镇签署茶产业合作协议，福安东元电机项目加快推进，霞浦与台湾顶新国际集团签订食品加工项目协议。全年实际利用台资245万美元，全市台资企业达95家，对台进出口贸易额达3407万美元，增长104.5%。

（三）着力民生民心，社会保持和谐稳定

惠民措施有效落实。20件21项为民办实事项目如期推进，其中17件18项为民办实事项目全面完成，3件3项跨年度项目达到即期目标。全年新增城镇就业2.51万人、下岗失业人员再就业1.03万人、农村劳动力转移就业4.25万人。基本医疗保障制度覆盖全市城乡居民，新型农村合作医疗参合率达87.9%，解决医改后关闭破产和困难企业职工参加城镇职工医保1.5万人。城乡居民最低生活保障基本实现应保尽保，被征地农民社保政策暂行意见正式出台。新建经济适用房1334套、城市低收入家庭廉租房1273套，发放廉租住房租赁补贴1000户。“造福工程”和滑坡险情村搬迁5553户25217人，完成灾后重建377户。福安、寿宁社会福利中心加快建设，新建和改扩建农村敬老院13所。援建四川省彭州市升平镇的6个项目相继投入使用。

社会事业协调发展。自主创新能力不断提高，新认定高新技术企业14家、市级企业技术中心20个，成功对接“6?18”项目468项、总投资56.1亿元。42项关键技术专项列入国家和省计划，大功率全数字电机、汽车混合动力系统、稳定型二氧化锆、大黄鱼种质保持利用与健康苗种培育等关键性技术取得突破。教育事业稳步发展，中小学校舍安全工程完成重建、改建8万平方米，高中阶段毛入学率、幼儿入园率分别达72.8%和89%，分别提高7个和2个百分点。宁德师专新校区和宁德职业技术学院建设步伐加快，宁德师专“专升本”通过国家高校设置评议委员会评议。市机关幼儿园建成投入使用。重大疾病防控和卫生应急处置能力继续增强，甲型H1N1流感疫情防控依法、科学、有序进行。宁德市医院开工建设，改造提升县级医院4所、乡镇卫生院17所、社区卫生服务中心2所。举办庆祝新中国成立60周年系列文化活动和闽东红军独立师成立75周年纪念活动，“中国木拱廊桥传统营造技艺”被联合国批准列入首批急需保护的非物质文化遗产名录，“2131”电影放映工程和农村书屋建设成效居全省各设区市前列。我市运动员在第十一届全运会上取得2金1铜的好成绩，市第三届运动会成功举办。低生育水平保持稳定，连续16年完成省下达的人口控制目标。人事、编制、统计、老区、民族、宗教、广播电视、新闻出版等工作取得新成效，档案、老龄、地方志、水文气象、残疾人事业、关心下一代等工作进一步加强。

生态环境保持良好。节能减排目标责任制有效落实，重点领域和企业节能减排工作取得新成效，重点污染源在线监测监控体系不断完善，全市生态环境质量继续位居全省前列。敖江古田段、闽江古田段、交溪、霍童溪、七都溪等重点流域整治扎实推进，石材加工企业整治成效明显，主要流域水质功能区和县级以上饮用水源水质达标率100%，敖江流域古田段水质浊度≤60%的比例达79.2%。县级以上饮用水源地整治建设项目基本完成，104个县乡饮用水源保护区基本建立监管长效机制。建成福安、古田、柘荣3个污水处理厂和周宁垃圾无害化处理场，城市内河水质明显改善。土地、矿产资源保护力度加大，连续11年实现耕地占补平衡。

“平安宁德”建设扎实推进。完善社会治安防控体系，人民群众对社会治安满意率达94.7%。矛盾纠纷排查调处不断深化，一批突出信访问题得到化解。安全生产“一岗双责”全面落实，重点行业、重点领域生产安全专项整治成效明显，全市安全生产形势保持稳定。应急管理体系逐步健全，应对突发公共事件能力逐步提升。国防动员、民兵预备役工作取得新成绩，圆满完成“闽东－2009”观摩活动。双拥、支前工作再上新台阶，荣获全省双拥模范城“三连冠”。人防、海防、反走私等工作取得新进展。

（四）民主法制和精神文明建设得到加强，依法行政水平进一步提高

各级政府依法接受人大及其常委会的法律监督、工作监督，坚持定期向人大及其常委会报告工作制度，依法执行人大及其常委会的决议决定，及时办理和反馈人大常委会开展执法检查、人大代表视察及专项调研提出的意见，认真办理人大代表建议、意见。全年共办理市人大代表建议236件，满意率达96.6%。自觉接受政协的民主监督，定期向政协通报情况，认真办理政协委员提案。全年共办理市政协委员提案337件，满意率达97.5%。加强与各民主党派、工商联、无党派人士和工会、共青团、妇联等人民团体的联系，广泛听取各界人士和人民群众的意见。大力弘扬“滴水穿石、人一我十、力求先行”的闽东精神，加强公民道德建设，扎实开展群众性精神文明创建活动，城乡群众文明素质逐步提升。“五五”普法深入开展。村（居）民委员会换届选举基本完成。清理整治非法买卖土地和违法违规建设、乱建坟墓、海上养殖取得阶段性成效。殡葬执法力度进一步加大，全市平均火化率达87.8%，提高13.4个百分点，超过省定指标7.8个百分点。规范市行政服务中心运作，已有45个部门492个项目实现网上审批，取消市级行政审批事项10项、下放16项、调整76项。推进政务公开和政府信息公开，深化效能建设、绩效管理，市政府与各县（市、区）政府签订的14个重点、难点专项工作责任状基本完成年度目标任务。政府投资项目监督管理有效加强，“小金库”和工程建设领域突出问题专项治理扎实开展。进一步完善行政执法监督，完成规范行政自由裁量权试点工作，依法办理行政复议案件，纠正损害群众利益不正之风，行政监察、审计监督取得新成绩。严格落实领导干部廉洁自律各项规定，源头防治腐败工作力度加大，廉政建设和反腐败工作不断加强。

各位代表，回顾过去一年的工作，我们取得的成绩来之不易。这是市委正确领导、科学决策，全市人民齐心协力、奋力拼搏，社会各界人士广泛参与、大力支持的结果。在此，我代表宁德市人民政府，向全市人民致以崇高的敬意，向给予政府工作有力支持和有效监督的人大代表、政协委员、各民主党派、工商联、各人民团体、无党派人士、离退休老同志和社会各界人士，向为宁德发展作出积极贡献的中央及省属驻宁单位、驻宁部队、武警官兵、公安民警、民兵预备役人员，向关心支持宁德发展的港澳台同胞、海外侨胞、国际友人，表示诚挚的感谢！

在肯定成绩的同时，我们也清醒地认识到发展还面临不少困难和问题，主要是：经济总量小，工业化水平低，产业规模不大，企业自主创新能力不强，节能减排压力较大，结构调整和发展方式转变还要付出更大的努力；基础设施仍然滞后，支撑大项目落地的水、电、路、排污等配套设施不够完善；市本级经济实力不强，中心城市辐射带动作用较弱；城镇化水平不高，县域经济发展动力和活力不足，城乡统筹还需要进一步推进；农民持续增收的基础还不稳固，农村生产生活条件尚需改善提升；教育、医疗等公共服务资源总体不足，分布不够均衡，与群众需求还有差距；安全生产仍有隐患，社会稳定还面临一些不确定因素；政府职能有待进一步转变，投资软环境还不尽如人意，一些干部思想不够解放、作风不够务实，缺乏干事创业激情，一些工作人员责任意识、服务意识不强，工作效率不高，甚至存在不作为、乱作为和消极腐败现象。对这些问题，我们将高度重视，采取更为有力的措施切实加以解决。

二、扎实做好2010年工作

今年是全面贯彻国务院《意见》的第一年，是深入推进环三都澳区域发展的重要一年，也是宁德撤地建市十周年。我们正处在前所未有的重要战略机遇期。国家鼓励东部地区率先发展、支持福建省加快建设海峡西岸经济区；省委、省政府支持环三发展，把环三区域列入海西重点培育的新增长区域；高速铁路通车进一步拉近了宁德与长三角、珠三角特别是福州、温州等发达地区的时空距离，宁德区位、港口、资源、对台和后发优势日益凸显。我们要坚定发展信心，抢抓发展机遇，把促进经济又好又快发展与加快经济发展方式转变有机统一起来，进一步解放思想，振奋精神，奋发图强，在新的起点上推进环三跨越发展。

今年政府工作的总体要求是：以邓小平理论和“三个代表”重要思想为指导，深入学习实践科学发展观，认真贯彻落实胡锦涛总书记在福建考察时的重要讲话精神，按照党的十七大和十七届三中、四中全会及中央、全省经济工作会议的部署要求，紧紧抓住国务院出台支持福建省加快建设海峡西岸经济区《意见》的重大历史机遇，全力推进环三都澳区域发展战略实施，在调整结构中加快发展，在转变方式中提升质量，在先行先试中拓展空间，在改革开放中激发活力，在统筹协调中促进和谐，在奋力争先中力求跨越，全面完成“十一五”规划目标任务，以崭新面貌迎接撤地建市十周年，以优异成绩推动海西东北翼中心城市迈上新台阶。

经济社会发展的主要预期目标是：地区生产总值增长14%左右，其中第一、第二、第三产业增加值分别增长4.5%、19%和13.5%；全社会固定资产投资增长20%；社会消费品零售总额增长17%；外贸出口增长15%；验资口径实际利用外商直接投资增长13%；财政总收入及地方级财政收入分别增长12%、13%；城镇居民人均可支配收入实际增长8%；农民人均纯收入实际增长7%；居民消费价格总水平涨幅控制在4%以内；城镇登记失业率控制在4.2%以内；人口自然增长率控制在8‰以内；单位生产总值能耗降低0.7%，化学需氧量排放下降1.1%，二氧化硫达标排放并努力控制排放量增长。在实际执行中，争取更好更快。

实现上述目标，重点要做好八个方面工作：

（一）着力巩固“三农”基础，大力发展现代农业

提高粮食保障能力。认真落实扩大涉农补贴规模、增加农机具购置补贴、提高主要粮食品种最低收购价等政策，充分调动农民种粮积极性。严格按照“稳面积、不抛荒、增产量”的要求，挖掘粮播面积潜力，大力推广增产实用技术，积极发展超级稻、再生稻，提高单产水平，确保粮播面积和产量稳定。落实储备定单收购政策，积极推进市、县中心储备粮库建设，切实保障粮食安全。

推进农业产业化经营。深入实施“百龙带动”工程，继续扶持壮大国家、省、市级农业产业化龙头企业，力争新增产值超亿元企业3家。加快发展农民专业合作社和农村行业协会，提高农业生产组织化程度。推广集约化、设施化、规模化、标准化生产，支持优势产区集中发展茶叶、水产、食用菌、林竹、果蔬、药材等特色产品，争创一批国家级、省级标准化示范区和示范基地。积极发展品牌农业，推行农产品质量认证和标识认证制度，进一步拓展“柘荣太子参”、“古田银耳”、“坦洋工夫”、“福鼎白茶”、“闽东大黄鱼” 等品牌效应。实施新品种、新技术、新肥料、新农药、新机具推广示范工程，推动“五新”融入生产、加工、流通各环节，构建支撑现代农业发展的技术链和服务链，促进增产增收。

加强农业农村基础设施建设。推进土地开发整理，加快中低产田改造、旱片整治和高标准农田建设。突出抓好民生水利工程建设，完成海堤除险加固29公里和小型水库除险加固37座，积极推进交溪流域上白石水利枢纽工程前期工作，解决15万以上农村人口安全饮水问题。硬化农村公路300公里，加快危桥改造和撤渡建桥工程建设。完成“造福工程”及滑坡险情村搬迁2.5万人以上。继续深入开展“农村家园清洁行动”，做好10个乡镇300个村庄垃圾治理，新建农村户用沼气池3890口。加快防汛抗旱基础设施建设，加强海洋、地震、气象、地质等灾害和森林火灾监测预警，有效防控动植物疫病、有害生物入侵和森林病虫害，提高预防和应对自然灾害能力。

深化农村体制机制改革。推进集体林权制度改革，完善配套政策，降低育林基金征收标准，扩大林权抵押贷款，做好屏南县林木采伐制度改革试点工作。鼓励金融机构拓展农村金融业务，改善农村信贷服务，组建福鼎市恒兴村镇银行，积极发展农村保险事业。加快供销合作社改革发展，完善现代经营服务和购销网络，增强基层社和联合社服务功能。继续做好社会主义新农村试点示范村和扶贫开发重点村建设。健全完善干部驻村任职、科技特派员、农村“六大员”等工作机制，加强乡镇“三农”服务中心、农村社区工作者队伍建设和农村实用人才培训。提高村主干待遇，市财政每月补助村主干100元。

（二）着力推动产业结构调整，促进经济发展方式转变

加快产业集聚壮大。积极对接省重点产业调整振兴实施方案，重点组织实施好总投资787亿元的30个产业振兴项目，扎实推进105个成长型工业新增长点项目和40个工业技改项目，确保工业增加值增长20%、规模以上工业增加值增长20%以上，努力打造海西临港先进制造业基地。传统产业要加大技改和研发投入，促进电机电器、船舶修造、食品加工、汽摩配件、医药化工等特色产业的升级改造，壮大集群规模，打造东南沿海重要的船舶工业、机电装备、机动车配件生产基地。石化产业要推进中海油海西宁德工业区建设，加快建设溪南半岛17平方公里围海造地工程，全力推进LNG接收站、油品储备、燃气发电厂、炼化一体化等项目前期工作，力争启动建设油品储备项目，建设新兴石化产业基地。能源产业要加快宁德核电一期工程、霞浦大京风电场和聚合物锂离子电池项目建设，推进大唐火电二期尽快上网商业运营，争取开工建设宁德火电三期，深化宁德核电二期工程、华能核电、大唐宁德煤炭集散中心、福鼎八尺门潮汐电站、周宁抽水蓄能电站等项目前期工作，建设东南沿海重要的能源基地。钢铁冶金产业要抓住省政府与鞍钢建立战略合作伙伴关系的机遇，加紧推进大型钢铁项目前期工作，争取早日落地，建设先进的冶金基地。加快镍合金、高纯锆、双氧水等项目建设，深化多晶硅、风电设备、豪华游艇等项目前期工作。

提升工业园区层次。各县（市）要整合现有工业园区，确定重点园区，明确园区产业、功能定位，引导相关企业、项目、研发中心、培训中心向园区集中，努力形成以主导产业企业为龙头、以专业化协作企业为基础的产业集群。市本级重点抓好海西宁德工业区、闽东工业园区和台湾工业园的建设。编制完成重点园区总体规划，上半年全面完成重点园区规划环评。建立多元化投资机制，完善工业园区水、电、路、信息、集中治污等基础设施和生活、教育、卫生配套设施，为园区发展提供优良的硬件保障。落实先进制造业用地扶持政策，建立工业用地价格调节机制。制定出台重点工业园区优惠政策和考核办法，考核结果作为政府绩效评估的重要内容。

加快推进自主创新。发挥福安“中国中小电机之都”、福鼎“中国化油器名城”等区域品牌优势，扩大现有工业驰名商标影响力，推动企业通过联合、兼并、重组进行品牌整合。继续实行创牌重奖政策，力争年内新增中国驰名商标2个。加强创新服务平台建设，充分发挥市高新技术创业服务中心和8个行业技术研发中心作用，抓好“6·18”项目对接和成果转化，做好重点工业科研和企业技术开发项目攻关，新认定高新技术企业10家、市级企业技术中心10个。以宁德市院士科技园为载体，规划建设市高新技术创业园，完善园区生活配套设施，引导各类人才、技术、项目向园区集聚。认真落实经济补助、职称评审、创业支持、户籍迁移、子女就学、家属就业、社会保险等政策，加快引进优秀人才。鼓励有条件的企业设立博士后科研流动站，推进人才柔性流动，培养和引进学术技术带头人、科技领军人才、一线创新人才和创新团队。

积极扶持企业发展。鼓励和引导企业优化重组，加快股份制改造步伐，力争完成2家以上企业股份制改造，为上市打好基础。对成功上市的企业，市、县两级各奖励100万元。积极向上争取各类扶持资金，综合运用财政贴息、税收政策等多种手段，加大中小企业扶持力度。加强担保机构规范管理，引导担保机构做大做强，鼓励金融机构开展以不动产、动产、权利为抵（质）押的信贷业务，有效缓解中小企业融资担保难题。

（三）着力扩大投资和消费，有效增强内需拉动

保持投资持续增长。继续开展“项目建设攻坚年”活动，确保完成年度全社会固定资产投资345亿元以上。全市计划安排重点建设项目118个，总投资1277亿元，年度计划投资189亿元，占全社会固定资产投资计划的54.8%。其中，在建项目100个，年度计划投资174亿元；预备项目18个，年度计划投资15亿元。计划建成投产或部分投产35个。加快宁德核电、宁武高速公路和海西宁德工业区启动项目等重中之重项目建设，争取完成投资额110亿元以上。深化项目前期，围绕中央、省扩大投资的基本方向和重点领域，从产业链延伸、产业目录细化、科技成果转化中生成项目，加快推进事关宁德长远发展、全局发展的15个重大项目前期工作。切实保障项目建设，健全完善重点项目领导责任、协调推进、跟踪服务和督查考核等工作机制，积极主动为重点建设的项目报批、资金争取、征地搬迁、技术难题、质量监控等提供更好更优的服务，确保项目顺利推进。

扎实开展“交通建设年”活动。大力推进交通通道建设，努力构建以港口为中心、北承南联、西进东出的现代化综合交通网络，打造东南沿海重要港口枢纽。围绕完善高速公路网，加快宁武高速公路宁德段建设进度，开工建设沈海复线蕉城至连江浦口段、京台高速公路宁德段、溪南疏港高速公路，深化沈海高速公路复线蕉城至福鼎段和三都岛连接线、福泰高速公路以及漳湾至古田等一批疏港高速公路的前期工作，为“十二五”实现县县通高速打下基础。围绕建设铁路网，加快建设宁德白马港铁路支线，开工建设合福铁路宁德段，力争明年动工建设衢宁铁路，抓紧合福铁路宁古支线以及溪南、漳湾等铁路支线前期工作。围绕完善港区集疏运条件，完成南起罗源鉴江、北至福鼎沙埕全线疏港通道规划，近期启动建设连接三都澳港、三沙港、沙埕港各作业区的疏港公路，中心城市动工建设罗源鉴江至漳湾码头疏港公路。基本完成霞浦盐田至溪南疏港二级公路主体工程。进一步推进三都澳水域扩大开放工作，加快建设三都澳港区城澳1＃、2＃多用途泊位，开工建设漳湾对台客货运码头，继续深化城澳、漳湾、白马、溪南等作业区业主码头和三都澳30万吨级深水航道一期工程前期工作。完善普通公路及重要路网，续建新建省级干线公路14条，基本完成宁古二级公路主体工程。力争年内动工建设宁德霞浦机场民航扩建工程。

努力扩大消费需求。认真落实义务教育学校、公共卫生和基层医疗卫生事业单位绩效工资政策，加大对低收入困难群体的补助力度，引导企业建立和完善职工工资正常增长机制，提高城乡居民的收入预期和支出预期。努力拓展农村消费，推进“万村千乡”和“双百”市场工程，改造提升城乡集贸市场和批发市场，大力发展食品、日用品、药品、农资等经营点和加盟连锁“农家店”，继续实施家电、摩托车下乡和汽车以旧换新，保证产品质量和售后服务。着力促进旅游升温，加快连接景区的公路、码头建设，鼓励发展星级饭店，大力开发旅游特色产品，积极引进境外、省外有实力的旅行社到我市开办合资或独资旅行社。扎实做好宁德世界地质公园迎接联合国专家现场考评各项准备工作，加快白水洋、太姥山创建5A级景区和鲤鱼溪、九龙漈创建4A级景区工作，推进三都澳、白云山、杨家溪、翠屏湖、东狮山、杨梅洲等景区建设，推动屏南县争创中国旅游目的地，打造海西特色文化和生态旅游胜地。支持居民自住和改善性住房消费，增加普通商品房供应，抑制投资性购房，保持商品房价格基本稳定。规范发展住房二级市场，盘活租赁市场。加强房地产市场监管，加大对圈地不建、捂盘惜售、囤积房源、哄抬房价等违规行为的查处力度。扶持现代物流业，依托港区、园区和城区，规划建设现代物流园区和物流中心，培育和引进一批覆盖范围大、功能作用强的专业化骨干物流企业。积极引进国际知名的大型超市和连锁经营龙头企业，确保东方伟业沃尔玛购物广场年底开业，加紧完善食用菌、水产品、太子参等专业批发市场，加快推进中国海峡大茶都二期工程以及电机电器、船舶修造等专业市场建设，着力培育连锁超市、电子商务等新型业态。发挥宁德会展中心作用，积极承办各类经贸洽谈、特色产品展示和专业性展会，提升专业办展水平。积极培育汽车销售、教育培训、医疗保险、体育保健、拍卖租赁、担保服务等新的消费热点，适应群众生活多样化、个性化的需求。整顿和规范市场秩序，抓好食品药品专项整治，保障群众放心购买、安心消费。加强价格监控，做好居民基本生活必需品的生产、调运和储备，确保供应量足、质优、价稳。

（四）着力深化改革开放，提升经济发展活力与动力

深化重点领域和关键环节改革。加快行政管理体制改革，推进市、县政府机构改革，深化乡镇机构改革。逐步建立国有资本经营预算管理、业绩考核、激励和约束等机制，抓紧组建市交通、能源、城市建设等区域性的国有资产集团，实现国有资产保值增值。落实促进民营经济发展的政策措施，吸纳民间资本参与国有企业改组改造、基础设施和社会事业项目建设，增强民营经济活力。推进用地、水权、水价、排污权交易等制度和电价综合改革，不断拓展公共资源市场化配置领域。强化财税收入征管，科学挖掘增收潜力，严格控制党政机关公用经费，加快重点支出和民生支出的进度。清理和统筹部门连续两年以上的项目资金，开展财政支出绩效评价试点工作，确保当年收支平衡。加强与开发性银行、进出口银行及商业银行的沟通合作，积极引进市外银行在我市直接设点营业，组建宁德农村商业银行，有效增加信贷投放。鼓励企业发行债券或在境内外资本市场融资。

推动对外贸易平稳回升。加快推进省电机产业出口基地和市船舶、水产品出口基地建设，鼓励出口企业加快技术升级与转化，加强出口产品研发，力争新增省级出口名牌企业10家。组织企业参加国内外重要展会，鼓励企业设立境外生产基地和营销网络，积极开拓东盟等自贸区市场，巩固欧美日等传统市场，拓展拉美、非洲等新兴市场。扩大出口企业出口退税先退后审和出口信用保险覆盖面，增加出口信用保险补贴。扶持外贸行业商协会，及时为企业提供相关政策、预警信息。

提高招商引资质量和水平。实行有备招商，重点筛选一批产业龙头项目和关键配套项目，完善项目战略策划，建立招商项目库和外商投资指导目录，鼓励外资投向产业调整振兴等重点领域，力争一批石化、钢铁、船舶修造、电机电器和现代流通等重点产业项目落地。注重招商质量，坚持有选择招商，强化与世界500强企业、央企、大型国企和上市公司等重要战略投资者的合作，积极引进投资强度大、科技含量高、产业效益优、生态友好型的项目和企业。创新招商方式，办好首届海峡两岸电机电器博览会暨第11届宁德投洽会和福建宁德·香港投资洽谈会，积极组织和参加上海世博会、“9·8”和“6·18”等重大活动。充分发挥宁德在外商会和温州宁德商会等商团的作用，加强园区招商、中介招商、网络招商和以商招商，支持现有外资企业增资扩产。实施“回归工程”，鼓励海内外乡贤回乡投资兴业。继续实施重点外资项目和重点外资企业挂钩责任制，强化签约项目跟踪服务，进一步提高项目报批率、动工率和到资率。

进一步扩大宁台交流合作。积极实施对台先行先试政策，努力打造海西对台交流合作的重要平台。完善宁德—基隆经济文化对口合作协调机制，尽快开通宁德港与基隆港的双向航线，推进宁马台客货运直航常态化。加快宁德台湾工业园和台湾商贸城建设，推进宁台产业深度对接，主动承接台湾电机电器、电子信息、新能源、光电等产业转移，促进一批台资先进制造业和高新技术项目生成落地。加快推进台湾水产品集散中心建设，鼓励农村合作经济组织、农业龙头企业与台湾工商会、农民协会加强协作，继续强化宁台乡镇对接，积极引进台湾先进制茶工艺等农产品精深加工技术，引导台商投资我市农业产业项目。积极融入“海峡旅游”品牌，精心设计海峡旅游精品线路，主动与台湾共享旅游资源、互推旅游市场，推进与基隆、马祖结成旅游友好合作区，把宁德打造成为海西东北翼对台旅游集散中心。充分发挥陈靖姑、圆瑛法师、甘国宝和畲族文化影响力，举办第二届海峡论坛两岸少数民族文化节，积极推动两岸社会力量、民间组织之间的交流，吸引更多的台湾同胞来宁寻根谒祖、探亲访友、投资兴业。

（五）着力加强生态环境建设，提高可持续发展水平

积极推进节能减排。强化节能减排评估审查，严把新上项目资源能耗关、环境保护关，坚决淘汰“十五小”、“新五小”等落后生产能力，发展低碳经济。加强合同能源管理和重点污染源在线监控，突出做好冶金、电力、化工等重点产业节能降耗和建材、制革、造纸等重点行业污染治理。完善市污水处理厂管网配套，动工建设宁德北区污水处理厂，深化东兰组团污水处理厂前期工作，加快建设市生活垃圾焚烧发电厂。建成周宁、屏南、寿宁污水处理厂和周宁、寿宁、柘荣垃圾处理场，实现每个县（市）有一个污水处理厂和一个垃圾处理场。强化节能监督和管理，在工业、建筑、交通、农村、商业、民用、政府机构等重点领域广泛开展节水、节电、节材、节地活动。积极推进循环型工业园区、农业园区和节约能源试点工程。

强化环境综合治理。继续依法整治敖江古田段、闽江古田段、交溪、霍童溪、七都溪和全市县、乡镇集中式饮用水源保护区，控制重点流域石板材加工和禽畜养殖污染，保障生活饮水水质安全。加强海洋环境监测，加大海上养殖综合整治力度，制定出台征收海域生态保护费政策，依法清理污染大、效益低的浅水养殖，科学调整养殖区范围，推广生态养殖技术，努力减少冰鲜饵料投放，有效改善海域生态环境。切实保护水源地及其周边生态环境，全面拆除水源保护区内生产性、经营性排污口及违章建筑，全面禁止水源保护区内采石、采砂、取土、开矿等违规行为，推进“青山挂白”治理。狠抓城市内河、噪音和机动车污染治理，控制农村面源污染，改善城乡生态环境。

合理开发利用资源。实行严格的岸线管制措施，全面清理圈而不建、未批先建的岸线、滩涂、土地、水域等资源。充分运用土地价格调节机制，引导节约集约用地。加快围海造地工程建设，积极推进土地开发整理，确保耕地占补平衡。抓好公路、铁路、主要流域两侧一重山和城区、景区、自然保护区等重点区域生态保护，推进生态公益林、湿地、沿海防护林保护和建设。推进矿产资源开发整合，规范勘查开发行为，全面停止审批新、扩、改建的矿山开采和石板材加工项目，力争每年矿山开采总量削减5%以上。

（六）着力城乡统筹，促进区域协调联动发展

推进绿色宜居海湾新城建设。围绕“临海、跨海、环海”三步跃升发展战略，编制完成环三都澳城市群规划，抓紧中心城市总体规划修编、滨海新城概念规划以及各类控制性详规、专项规划编制。启动东侨设立建制区和蕉城郊区乡镇改设街道办事处工作。加快推进蕉城漳湾、金涵等片区建设，继续推进东侨新区提升拓展，延伸宁德中心城市南北临海两翼。启动闽东工业园区扩展规划和宁德台湾工业园控制性详规编制，全力推进园区征地，加快水、电、路、集中治污等基础设施和学校、医疗、餐饮、休闲等配套服务设施建设，提升园区层次。积极引进项目落户园区，引导市区现有企业逐步迁入园区，增强中心城市集聚能力。加快推进城市基础设施建设，建成五星级宁德会展酒店续建工程、东湖公园木栈道、火车站站前广场以及金马北路、福宁北路、学院路二期等城市路网，完成环城北路改造工程和鹤峰路综合整治，确保9月份动工建设闽东中路主体工程，完善镜台山、南漈山公园设施，继续做好东湖清淤、金溪流域综合整治。实行中心城市管理重心下移，做到守土有责、管理有章、执法有权，切实加强城乡规划执法监察，强化以交通秩序、环境卫生、市容市貌、市场秩序等为重点的城市综合管理，逐步改善城市面貌，争创省级卫生城市、园林城市、先进文明城市。

推进县域经济发展。引导各县（市）主动对接环三发展规划，进一步调整、修改和完善区域内各项规划。做好县域城区旧城改造和新区建设，鼓励发展大型商贸、星级酒店等城市服务业，抓紧实施一批公共交通、城市垃圾、污水处理项目，合理布局城区教育、文化、卫生资源，进一步搞活经济、集聚商气、带旺人气。引导各县（市）立足各自资源优势、产业优势和区位优势，积极培育特色经济，打造一批优势明显、带动力强的工业园区板块、特色产业板块和现代农业示范园板块。推进山区和沿海开展经贸、教育、农业、旅游和基础设施建设等合作，积极引导产业共建，努力在飞地政策实施上实现突破，促进山海协调发展。

推进城镇化进程。按照打造“规划先行、功能齐备、设施完善、消费便利、保障一体”的宜居城市综合体要求，在做好福安赛岐、福鼎秦屿省级重点小城镇综合改革建设试点的同时，每个县（市、区）也要推出1－2个乡镇作为试点。修订和完善小城镇总体规划和土地利用总体规划；由市政府牵头，蕉城、福安、霞浦、福鼎政府配合，编制完成三都澳内及霞浦、福鼎沿海和高速公路沿线的城镇总体规划。加大小城镇基础设施建设投入，鼓励支持房地产开发企业参与小城镇建设，合理安排区域性基础设施和公共服务设施，提高教育、医疗、文化、体育等基本公共服务能力。积极稳妥解决农民工在城镇就业和生活问题，逐步实现农民工在劳动报酬、子女就学、公共卫生、住房租购以及社会保障方面与城镇居民享有同等待遇。放宽中心城镇落户条件，使在城镇稳定就业和居住的农民有序转变为城镇居民。抓紧做好高速公路、铁路沿线旧村改造规划。

推进区域协作联动。发挥海西二十城市协作机制和闽浙赣皖福州经济协作区、闽东北五市协作区等跨省市协作组织的作用，建立更加紧密的区域合作机制，强化区域发展规划、专项规划的相互衔接，促进产业协作配套、设施共建共享和生态协同保护，实现城市及区域资源要素优化配置。积极承接长三角、浙西南地区产业转移，推进与江西等中部内陆地区合作，促进港口建设和物流业发展，加快建成海西对接长三角的前沿区域。加强与港澳地区的经贸合作，大力维护归侨侨眷、海外侨胞合法权益，吸引港澳同胞、海外侨胞到我市投资兴业。积极做好对口支援，全面完成援建四川彭州任务。

（七）着力保障和改善民生，让人民群众共享改革发展成果

千方百计扩大就业。实施更加积极的就业政策，统筹做好高校毕业生、复员转业军人等群体就业工作，招募高校毕业生服务社区，力争全年城镇新增就业2.45万人。继续实施“一户一工一技能”工程，积极做好困难弱势群体、返乡农民工和城镇下岗失业人员培训与就业安置，加强农村劳动力技能培训，引导农民有序外出就业，鼓励农民就近转移就业，完成农村劳动力技能培训2.5万人，新增农村劳动力转移就业3.33万人。健全协调劳动关系三方机制，推动和谐企业建设，构建和谐劳动关系。

完善城乡社会保障。积极扩大“五险”覆盖面，突破非公经济组织从业人员、城镇个体工商户、灵活就业人员和农民工参保，提高社会保障水平。推动养老保险无障碍转移接续，积极稳妥推进事业单位养老保险制度改革，认真解决未参保集体企业退休人员基本养老保障和其他社会保障遗留问题，在福鼎市开展新型农村社会养老保险试点。加大临时救助制度建设，将城乡低收入家庭中60周岁以上老年人、革命“五老”、重症患者纳入城乡医疗救助范围。建成廉租房1313套，规划建设市本级保障性住房、安置房2000套。加大农村危房改造支持力度，改善中低收入住房困难家庭居住条件。继续开展老区工作优先行动，支持革命老区、少数民族地区、海岛等欠发达地区和库区移民安置区基础设施建设，在政策、资金、项目等方面给予倾斜。发展社会福利事业，启动建设市社会福利中心。加强老龄工作，切实维护和保障妇女儿童合法权益，积极发展慈善事业和残疾人事业。

统筹社会事业发展。坚持优先发展教育，巩固提升“双高普九”质量和水平，解决好困难家庭子女上学问题，推进中小学校舍安全工程、寄宿制学校建设和城镇中小学扩容，抓好87所义务教育学校标准化建设，进一步规范中小学办学行为，促进义务教育均衡发展。建成宁德学院新校区巨构体、学科院系楼、学生宿舍楼、学生食堂、运动场以及校区路网等设施，完成宁德师范附小教学综合楼重建工程，确保9月份新生入学。加快宁德职业技术学院、市委党校校区建设。大力发展职业教育，加强中等职业学校基础能力建设，推进县级职业教育资源整合，改善县级职业教育中心办学条件，建立急需紧缺人才培养培训基地和区域性职业教育实训基地，增强职业教育为区域经济和社会发展服务的能力。积极关心幼儿教育，加强民办幼儿园规范管理。扎实推进普通高中、特殊教育、民族教育和终身教育发展。推进医药卫生体制改革，健全完善市、县、乡三级疾病预防控制体系、突发公共卫生事件应急机制和医疗救治体系，加强疾病控制、卫生监督、妇幼保健等公共卫生服务，做好甲型H1N1流感等重大疾病防控和救治工作。继续完善新农合制度，探索开展城镇居民医疗保险门诊费用统筹。加快宁德市医院建设，加强农村医疗卫生机构基础设施和社区卫生服务机构标准化建设。完善医患纠纷预防和处置长效机制，构建和谐医患关系。实施“文化惠民工程”，推进文化馆、文化广场等公共文化基础设施建设，做好市大剧院、图书馆、博物馆前期工作，继续实施乡镇综合文化站新改扩建、文化信息资源共享工程、农家书屋建设工程，全面推进有线数字电视整体转换，继续做好农村数字电影放映工作。大力推进文艺精品创作，深入开展丰富多彩的群众文化活动。加强重点文物保护、文化典籍整理和保护以及非物质文化遗产的传承和保护工作。做好畲族文化园规划，争取设立闽东畲族文化生态保护实验区。启动建设市体育中心二期工程，积极开展全民健身活动、老年体育活动，继续实施农民体育健身工程，全力备战第十四届省运会。开展第六次全国人口普查。加强人口计生公共服务网络体系建设，实施优生促进工程，有效解决出生人口性别比、计生技术服务、流动人口计生管理等难点，持续稳定低生育水平，不断提高出生人口素质。

（八）着力加强民主法制和精神文明建设，促进社会和谐发展

加强民主法制建设。认真贯彻落实监督法及福建省实施办法，坚持向人大及其常委会报告工作制度，依法接受人大及其常委会的法律监督、工作监督，依法执行人大及其常委会的决议决定，认真办理人大代表建议、意见，做好人大代表执法检查、视察等活动成果的采纳和落实。坚持定期向政协通报情况，主动接受政协的民主监督，支持政协开展调研和视察活动，认真办理政协委员提案。虚心听取各民主党派、工商联、无党派人士和各人民团体的意见建议，主动接受新闻舆论和社会公众监督。做好“五五”普法总结验收工作，深入推进普法依法治理。认真贯彻落实依法行政实施纲要和加强市县政府依法行政的决定，加强对行政权力的制约和监督，规范行政权力运行和行政自由裁量权。依法依规清理非法买卖土地和违法违规建设、乱建坟墓和海上非法养殖，进一步提高政府公信力和宁德对外形象。

加强和改进社会管理。健全基层社会管理体制，推进城乡和谐社区建设。加强社会治安综合治理，推进新一轮“平安宁德”建设，严密防范和依法打击违法犯罪活动，集中整治治安突出问题和网络安全问题，不断增强人民群众安全感。推进社会矛盾化解，依法依规处理重点信访、越级上访、老信访问题和群体性事件，认真处理好征地搬迁、企业改制、环境污染、劳资纠纷、涉法涉诉等方面的矛盾和问题，努力把不稳定因素化解在基层、化解在萌芽状态。严格落实“一岗双责”和企业主体责任，深入开展安全生产专项整治，加大事故隐患整改力度，最大限度地预防和减少安全事故发生。推进应急管理体系建设，提高突发公共事件信息报送的时效和质量，及时有效处置各类突发公共事件。

加强精神文明建设。以加强社会主义核心价值体系建设为根本，积极推进思想道德建设，扎实开展群众性精神文明创建活动，积极传承闽东红土地文化，大力弘扬“滴水穿石、人一我十、力求先行”的闽东精神。全面落实党的民族宗教政策，依法管理宗教事务，切实维护民族团结。巩固发展军政军民团结，加强民兵、预备役建设和人防、海防、反走私工作，深化爱民固边战略，妥善安置转业退伍军人，不断提高双拥共建水平，争创全国双拥模范城“三连冠”。认真做好老干部工作，积极做好新形势下的侨务、外事工作，继续做好人事、编制、审计、统计、档案、地震、地方志、水文气象、关心下一代等工作。

三、建设对人民负责的政府

面对新形势新任务，我们要不断加强政府自身建设，着力提高政府工作水平和工作能力，以有效作为更好地对人民负责、为人民服务、让人民满意。

解放思想，先行先试。各级政府要进一步解放思想、更新观念，坚持不懈地用科学发展观武装头脑、指导工作，扎实开展“经济发展方式转变年”活动，突出战略重点，抢抓战略机遇，坚定不移调结构，更大力度抓创新，切实把转变经济发展方式体现在加快发展、绿色增长、惠及民生上，抢占新一轮竞争的制高点，推动宁德在新的起点上跨越发展。进一步精心谋划、提升思路，锲而不舍地贯彻实施好环三发展战略，更新发展思路，确定更高目标，敢于突破，敢于超越，高站位、高层次、高水平编制“十二五”规划，只要是有利于推动发展的，要视野更阔一些，办法更多一些，胆子更大一些，步子更快一些。要着眼后国际金融危机时期的新变化，深入研究重大战略性课题，用足用好政策，千方百计从政策中生成项目、争取资金，下大力气做好重大项目前期工作，积极争取重大项目和生产力布局纳入国家、省规划。进一步提振精神、锐意创新，把改革创新精神贯穿于政府工作的各个环节，鼓励支持一切有利于发展的创新创造活动，支持先行探索者，宽容先试失误者，鼓励实践成功者，激发广大干部干事创业激情，努力形成勇于创新、勇于创造、勇于创业的浓厚氛围。

强化责任，求真务实。各级政府领导干部要尽心尽责、亲历亲为，把心思用到干事业上，把功夫下到察实情、出实招、办实事上，一级做给一级看，不搞形式主义、不做表面文章。对一些事关全年目标任务完成、事关长远发展、事关民心民生的大事、难事，要亲自协调、亲自落实，一竿子插到底，有效解决问题，确保各项工作强势推进。要层层落实、实干实效，定下来的事情要雷厉风行、抓紧实施，部署的工作要加强督查、一抓到底，一步一个脚印地推进各项工作，确保既定的目标和任务转变成工作的业绩和发展的成效。今年继续实行市政府与各县（市、区）政府集中签订重点专项工作责任状，加大督查督办力度，年终集中统一考评通报。要攻坚克难、强力突破，抓好资金筹措、征地搬迁、项目审批、软环境建设等瓶颈问题的解决和具体事情的处理，用最大的精力、最强的干部、最有效的方法，全力推进，力求实效。

勤政廉政，树立形象。优化政府服务，规范行政服务中心运行，完善各类办事公开制度，推进网上审批和电子监察，进一步转变政府职能。强化效能建设，推行绩效管理，完善民主评议政风行风制度，严格执行领导干部问责制，进一步治懒治庸，提高政府执行力。加强廉政建设，坚持用制度管权、管事、管人，强化对重点部门、重点事项和重点岗位的监督，加大财政资金和重大项目的审计和稽查，坚决治理和纠正损害群众利益的突出问题和不正之风，保证人民赋予的权力始终用来为民谋利益。

各位代表，全面完成“十一五”规划目标任务，实现对全市人民的庄严承诺，任务艰巨，责任重大。让我们紧密团结在以胡锦涛同志为总书记的党中央周围，在省委、省政府和中共宁德市委的坚强领导下，团结带领全市人民，解放思想、开拓创新，凝心聚力、力求跨越，全面完成“十一五”规划各项任务，为全力推进环三都澳区域发展和海西东北翼中心城市建设而努力奋斗！
